# 25-Hydroxyvitamin D, Vitamin D Binding Protein and Gestational Diabetes Mellitus: A Two-Sample Mendelian Randomization Study

**DOI:** 10.3390/nu16162603

**Published:** 2024-08-07

**Authors:** Yiwen Qiu, Diliyaer Ainiwan, Ye Huang, Libi Zhang, Haoyue Cheng, Xialidan Alifu, Haibo Zhou, Nuo Xv, Boya Wang, Shuhui Wang, Zexin Chen, Hui Liu, Danqing Chen, Yunxian Yu

**Affiliations:** 1 Department of Public Health and Department of Anesthesiology, The Second Affiliated Hospital of Zhejiang University School of Medicine, Hangzhou 310009, China; yiwenqiu@zju.edu.cn (Y.Q.); 22218236@zju.edu.cn (D.A.); 22218854@zju.edu.cn (Y.H.); 15313710752@163.com (L.Z.); 3150101365@zju.edu.cn (H.C.); 3130100017@zju.edu.cn (X.A.); 11918158@zju.edu.cn (H.Z.); 22318848@zju.edu.cn (N.X.); 22318872@zju.edu.cn (B.W.); 3190100548@zju.edu.cn (S.W.); 2 Department of Epidemiology and Health Statistics, School of Public Health, School of Medicine, Zhejiang University, Hangzhou 310058, China; 3 Center of Clinical Epidemiology and Biostatistics, Department of Scientific Research, The Second Affiliated Hospital of Zhejiang University School of Medicine, Hangzhou 310009, China; chenzexin@zju.edu.cn; 4 Sir Run Run Shaw Hospital, School of Medicine, Zhejiang University, Hangzhou 310016, China; lhui2010@zju.edu.cn; 5 Department of Obstetrics and Gynecology, Women’s Hospital, School of Medicine, Zhejiang University, Xueshi Rd #1, Hangzhou 310006, China; chendq@zju.edu.cn

**Keywords:** Vitamin D, 25(OH)D, Vitamin D binding protein, gestational diabetes mellitus, Mendelian randomization

## Abstract

Background: Numerous studies have examined whether vitamin D is associated with gestational diabetes mellitus (GDM). Nevertheless, it is still challenging to determine the causality, due to a number of shortcomings in observational research and randomized controlled trials. Objective: Mendelian randomization (MR) with two samples was conducted to investigate the potential causative association between 25-hydroxyvitamin D (25(OH)D), vitamin D binding protein (VDBP) and GDM risk. Methods: Publicly accessible summary data from independent cohorts were used for two-sample MR. For 25(OH)D, we obtained data from UK Biobank, IEU and EBI, then performed a meta-analysis to enhance the statistical power (via METAL); for VDBP, data were obtained from the INTERVAL study; for GDM, data were obtained from FinnGen. The inverse variance weighted (IVW) approach was performed as the main analysis, together with several sensitivity analyses, such as MR–Egger, maximum likelihood, weighted median, and weighted mode. Results: The IVW results revealed a weak negative causal connection between 25(OH)D and GDM risk [OR (95% CI) = 0.71 (0.50, 0.99), *p* = 0.046]. However, the causal association was unstable according to sensitivity analyses, and Cochran’s Q test revealed significant heterogeneity. After removing BMI-related IVs, the causal association between 25(OH)D and GDM disappeared [OR (95% CI) = 0.76 (0.55, 1.06), *p* = 0.101]. In addition, our study found no proof to support the assumption that VDBP level was related to GDM risk causally [OR (95% CI) = 0.98 (0.93, 1.03), *p* = 0.408]. Conclusions: According to this study, a weak negative causal association between 25(OH)D and GDM risk was found, while we had little proof to support the link between VDBP and GDM. To further explore whether total or free 25(OH)D levels and GDM are causally related, GWAS data with an emphasis on women of reproductive age and other ethnic groups are required.

## 1. Introduction

Vitamin D is a kind of essential steroid hormone, which is crucial for human growth and development [1]. To become physiologically active, vitamin D must go through two hydroxylation processes after it is consumed or produced. The first hydroxylation occurs in the liver, converting vitamin D to 25-hydroxyvitamin D (25(OH)D), the second hydroxylation occurs primarily in the kidney, converting 25(OH)D to its active form, 1,25-dihydroxyvitamin D (1,25(OH)_2_D), and then it combines with vitamin D receptor (VDR) to exert its effect [2]. In a range of metabolites, 25(OH)D is employed as the most important clinical biomarker of vitamin D status. Up to date, numerous studies have discovered a connection between low levels of 25(OH)D in pregnant women and a series of unfavorable perinatal outcomes, especially gestational diabetes mellitus (GDM) [3]. According to a meta-analysis published recently, pregnant women with vitamin D deficiency had a 26% higher risk of developing GDM than those with normal vitamin D concentrations [4].

Vitamin D binding protein (VDBP) is the main vitamin D carrier protein, encoded by the *GC* gene and formerly recognized as the group-specific component of serum (GC-globulin). Previous studies also demonstrated a positive genetic causal relationship between VDBP levels and serum 25(OH)D levels [5]. The concentration of VDBP will increase sharply during pregnancy, leading to a drop in the quantity of free vitamin D. Therefore, rather than assessing 25(OH)D alone, some research hypothesizes that taking into account both VDBP and 25(OH)D at the same time may be more reflective of the vitamin D status during pregnancy [6]. In addition, results from a cohort study reported that *GC* rs16847024 and *GC* rs3733359 were linked to a higher GDM risk [7].

However, whether or not these associations are causal is unknown. Additionally, residual confounding may be an explanation, which is plausible in observational investigations of incident GDM. Clinical trial results have demonstrated a conflicting impact of supplementation with vitamin D on the incidence of GDM but, due to problems with dosage, calcium combination therapy, compliance, and generalizability, careful interpretation of these findings is advised [8,9,10].

In order to infer the causality, Mendelian randomization (MR) is utilized to analyze genetic variation. Using instrumental variables (IVs), MR can investigate possible causative relationships between exposures and outcomes, limit the impact of confounders and other variables, and improve the validity of the causal evidence [11]. In addition, compared to traditional methods, the course of illness or reverse causality will not impact the results of an MR analysis. Therefore, in order to explore the potential causative association between vitamin D and VDBP with GDM, two-sample MR analysis was conducted in this study utilizing pooled data from large-scale open access genome-wide association studies (GWAS).

## 2. Methods

### 2.1. Study Design

**Figure 1:**
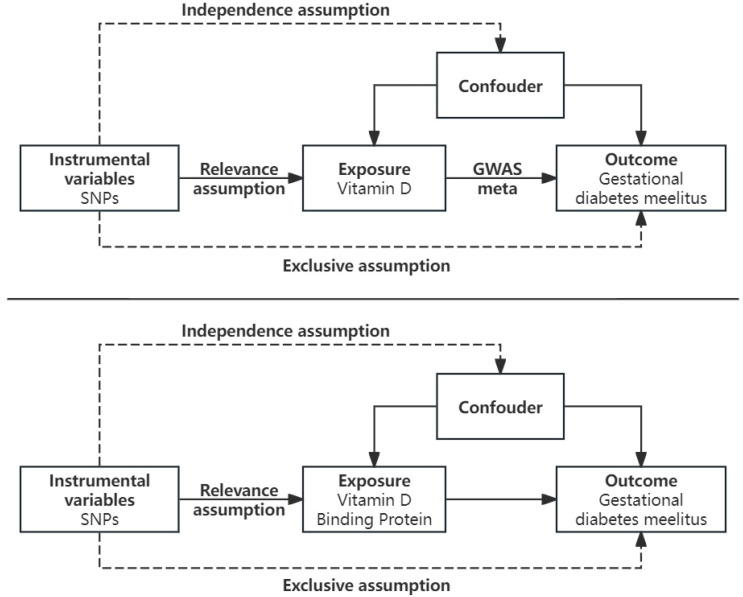
Schematic overview of the study design. In MR analyses, genetic variants must satisfy three principal assumptions to be legitimate instrumental variables (IVs). (1) Relevance assumption, i.e., IVs should be strongly associated with the exposure; (2) Exclusivity assumption, i.e., IVs should be associated with the outcome only through exposure; (3) Independence assumption, i.e., IVs should not be associated with confounding factors. SNP, single-nucleotide polymorphism.

The investigation of the causality between the exposures (VDBP and 25(OH)D) and the result (GDM) was conducted using two-sample MR. The following three assumptions were required to be met while selecting single nucleotide polymorphisms (SNPs) as IVs: (1) Relevance assumption, i.e., IVs ought to be closely linked to the exposure; (2) Exclusivity assumption, i.e., IVs ought to be connected with the outcome solely via exposure; (3) Independence assumption, i.e., IVs ought not to be linked to confounding factors (Figure 1).

### 2.2. Data Sources

The list of GWAS used in this study is shown in Table 1. The GWAS data on 25(OH)D were sourced from three datasets: (1) UK Biobank (UKB): 449,835 European participants (ID: ukb-d-30890_irnt); (2) European Bioinformatics Institute (EBI): 496,946 European participants (ID: ebi-a-GCST90000618); (3) Integrative Epidemiology Unit (IEU): 441,291 European participants (ID: ieu-b-4808). The MRC-IEU GWAS project contains all of these datasets. According to previously published literature, a chemiluminescent immunoassay was utilized to quantitatively determine the 25(OH)D level, and the unit of 25(OH)D level is nmol/L [12,13]. The GWAS data for VDBP level were sourced from the INTERVAL study, with 3301 European individuals [14].

The most current R9 edition of the FinnGen consortium provided the GWAS data on GDM, which were also accessible on the MRC-IEU public database (ID: finn-b-GEST_DIABETES). This database encompasses 5687 participants with a history of GDM and 117,892 controls, all from the Finnish population between the years 1970 and the present. GDM diagnosis is based on data from nationwide health registers (mainly using classification codes from the International Classification of Diseases) [15].

No specific ethical permission was needed because the data came from GWAS summary statistics that were made available in public databases. 

### 2.3. Genetic Variant Selection

The subsequent selection criteria were used to define the SNPs as IVs. (1) For genome-wide significance, a statistically significant threshold (*p* < 5 × 10^−8^) was established in order to guarantee that the SNPs were substantially connected with exposure. The *p* value cutoff would increase to 5 × 10^−6^ if the number of IVs were less than 20 [16]. (2) Excluded from the clump algorithm were SNPs with linkage disequilibrium (LD) (r^2^ > 0.001) inside a 10,000 kb clump window. (3) For each SNP, R^2^ and F-statistics were calculated (Formulas (1) and (2)) to evaluate the explained variance and potential weak instrument bias. The total R^2^ was calculated by adding the R^2^ values of all the SNPs. In Formula (1), the effect of allele frequency is indicated by EAF, the effect size of the SNP is represented by β, and the GWAS sample size is denoted by n. SNPs that correspond to a weak instrument bias are excluded when the F-statistic is less than 10. (4) Within the population, mutations are found to be more common than one percent of the total. (5) We used LD trait (https://ldlink.nci.nih.gov/?tab=ldtrait) (accessed on 22 April 2024) to verify the independence of each SNP selected in the earlier stage in order to make sure the IVs were free of confounders, and removed those associated with the confounder (body mass index, BMI) [17].

(1) $$R^{2}=2\times \left(1-\mathrm{EAF}\right)\times \mathrm{EAF}\times \beta^{2}$$

(2) $$F=\frac{R^{2}}{1\;-\;R^{2}}\times (n-2)$$

To enhance the statistical power of GWAS, we performed a fixed-effect inverse variance weighted (IVW) meta-analysis on the 25(OH)D-related IVs from three cohorts using METAL software (http://www.sph.umich.edu/csg/abecasis/Metal) (accessed on 22 April 2024). Based on the direction of effect and *p* value found in every study, METAL generates a signed Z-score. The sum of the Z-scores for each IV across studies is weighted, with each study’s weight determined by taking the square root of its sample size [18]. 

### 2.4. MR Analysis

We utilized the random-effect IVW as the main method, and MR–Egger, maximum likelihood, weighted median, and weighted mode were further employed to test the robustness of the primary finding [19,20]. The IVW findings are usually correct in the absence of horizontal pleiotropy [21]. When pleiotropy is present, the MR–Egger approach yields a regression slope that indicates the causal estimate and an intercept that indicates the presence of directional pleiotropy [19]. In addition, if there is no heterogeneity and pleiotropy, the maximum likelihood approach can also yield unbiased results [22]. When more than fifty percent of the IVs are invalid, both the weighted mode and the weighted median approaches can yield reliable estimates [23]. The results and stability of MR analysis were shown using scatter, forest, and funnel plots. For testing heterogeneity, MR–Egger intercept analysis and IVW were used to generate the Cochran Q-statistic; for testing pleiotropy, MR–PRESSO and MR–Egger intercept analysis were used. Furthermore, to evaluate the robustness of the results, a leave-one-out sensitivity analysis was carried out, in which each unique SNP was eliminated one at a time [16].

All statistical analyses were performed using the ‘TwoSampleMR’ packages (version 0.5.11), ‘MRPRESSO’ packages (version 1.0), and ‘LDlinkR’ packages (version 1.4.0) in R (version 4.3.3). Statistical significance was determined as a *p* value less than 0.05. 

## 3. Results

### 3.1. Causal Relationship between 25(OH)D and GDM

**Figure 2:**
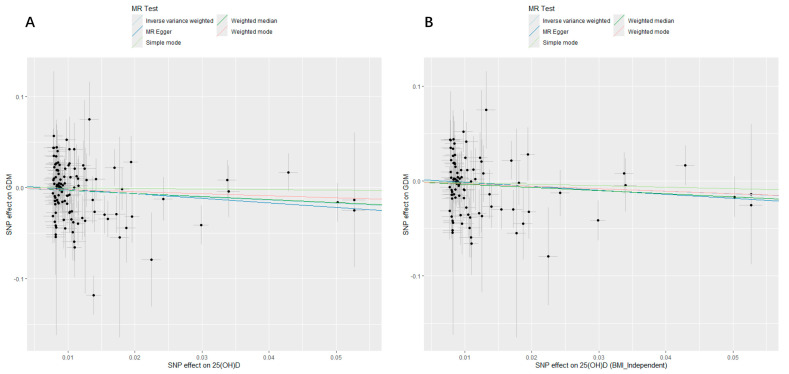
Scatter plots of MR analyses evaluating the causal effect of 25(OH)D on GDM. (**A**) Analysis of 25(OH)D and GDM. The X-axis shows the effect and SE for each selected SNP from the GWAS dataset of 25(OH)D. The Y-axis shows the effect and SE for each of these SNPs on GDM from the GWAS dataset of GDM. (**B**) Analysis of 25(OH)D and GDM with IVs independent of BMI. The X-axis shows the effect and SE for each selected SNP independent of BMI from the GWAS dataset of 25(OH)D. The Y-axis shows the effect and SE for each of these SNP on GDM from the GWAS dataset of GDM. MR, Mendelian randomization; GDM, gestational diabetes mellitus; SNP, single-nucleotide polymorphism; GWAS, genome wide association study; SE, standard error; IV, instrumental variable; BMI_Independent, selected IVs independent of BMI.

Table 2 and Table 3 show the MR analysis and sensitivity analysis of the causality between 25(OH)D and GDM, respectively. In the upper part of Table 2, 25(OH)D was causally negatively associated with GDM [IVW, OR (95% CI) = 0.71 (0.50, 0.99), *p* = 0.046]. The result of MR analysis in the maximum likelihood method was consistent with the primary IVW result [OR (95% CI) = 0.70 (0.52, 0.95), *p* = 0.022], and analysis outcomes of other MR approaches were similar, but did not reach statistical significance (all *p* > 0.05). To see the impact magnitude of each MR approach, a scatter plot was created, exhibiting a weak negative association trend of 25(OH)D and GDM (Figure 2A). In Table 3, the Cochran’s Q test revealed significant heterogeneity of MR analysis results (IVW, Q = 142.35, *p* = 0.021; MR–Egger, Q = 141.87, *p* = 0.019), which the leave-one-out analysis plot also helped to visualize (Figure S1A), indicating that 25(OH)D-related genetic tool variants may generate GDM via different possible routes. No pleiotropy was found with the MR–Egger Intercept test (Intercept = 0.003, SE = 0.005, *p* = 0.5465), but one pleiotropic outlier was found by using the approach of MR–PRESSO (SNP: rs12775091). In addition, to display each SNP estimate of the findings, a forest plot was created (Figure S2A), while symmetrical funnel plots showed no signs of selection bias (Figure S3A).

Thus, we reassessed the causality between genetically predicted 25(OH)D and GDM subsequently, with IVs independent of BMI, and the association is no longer significant in the lower part of Table 2 [IVW, OR (95% CI) = 0.76 (0.55, 1.06), *p* = 0.101]. No heterogeneity was found with Cochran’s Q test (IVW, Q = 95.01, *p* = 0.312; MR–Egger, Q = 94.54, *p* = 0.298) (Table 3) or the plot of the leave-one-out analysis (Figure S1B). No pleiotropy was found with the MR–Egger intercept test (*p* = 0.5108) or MR–PRESSO (Table 3). The scatter plot of the MR analysis evaluating the causative association between 25(OH)D and GDM with IVs independent of BMI is shown in Figure 2B, while the forest and funnel plots are shown in Figures S2B and S3B.

### 3.2. Causal Relationship between VDBP and GDM

**Figure 3:**
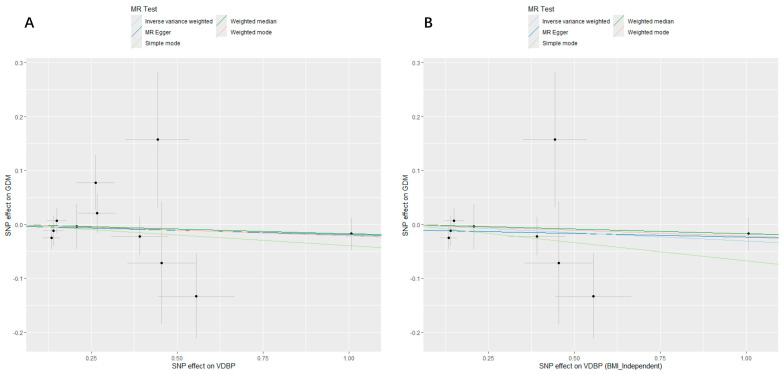
Scatter plots of MR analyses evaluating the causal effect of VDBP on GDM. (**A**) Analysis of VDBP and GDM. The X-axis shows the effect and SE for each selected SNP from the GWAS dataset of VDBP. The Y-axis shows the effect and SE for each of these SNPs on GDM from the GWAS dataset of GDM. (**B**) Analysis of VDBP and GDM with IVs independent of BMI. The X-axis shows the effect and SE for each selected SNP independent of BMI from the GWAS dataset of VDBP. The Y-axis shows the effect and SE for each of these SNP on GDM from the GWAS dataset of GDM. MR, Mendelian randomization; VDBP, vitamin D binding protein; GDM, gestational diabetes mellitus; SNP, single-nucleotide polymorphism; GWAS, genome wide association study; SE, standard error; IV, instrumental variable; BMI_Independent, selected IVs independent of BMI.

Table 4 and Table 5 show the primary MR analysis and sensitivity analysis of causal relationship between VDBP and GDM, respectively. As shown in Table 4, there was insufficient proof to support the link between VDBP and GDM, whether considering the confounder (BMI) or not [IVW, OR (95% CI) = 0.97 (0.92, 1.02), *p* = 0.243], and the other MR analysis techniques showed similar results (all *p* > 0.05). The effect and SE of each selected SNP on exposure and outcome were visualized in scatter plots (Figure 3). In Table 5, the SNPs did not show any discernible heterogeneity, according to Cochran’s Q statistics (all *p* > 0.05), and the plot of the leave-one-out test revealed the same finding (Figure S4). In addition, MR–Egger intercept analysis detected no horizontal pleiotropy (all *p* > 0.05), and the MR–PRESSO analysis revealed no outlier SNPs, indicating that the results of this MR study are solid and trustworthy, since it is highly unlikely that they would be impacted by plausible confounding pathways. The leave-one-out test, funnel plot, and forest plot all showed that each SNP’s distribution and effect are balanced (Figures S5 and S6).

## 4. Discussion

We investigated the possible causality of genetically determined 25(OH)D, VDBP and GDM using two-sample MR, respectively, with robust IVs from several European population-based large-sample GWAS databases. There was a weak inverse association between genetically predicted 25(OH)D level and GDM risk. The causality, however, was erratic and vanished when the BMI-related IVs were eliminated. In addition, the association between the risk of GDM and VDBP level was not well supported by the findings in our study.

There have been numerous epidemiological studies exploring the association between 25(OH)D levels in pregnant women and GDM risk, yet the conclusions remain controversial [24]. The previous study by our group found that vitamin D deficiency (25(OH)D < 20 ng/mL) in mid-pregnancy increased the risk of GDM by 44% [OR (95% CI) = 1.44 (1.12–1.86)] [25]. The meta-analysis made by Milajerdi A et al. [4] also pointed out that compared to pregnant women with appropriate vitamin D concentration, those with insufficient or deficient levels had a 26% increased chance of having GDM. However, a study conducted by Tkachuk et al. [26] among Russian pregnant women discovered no correlation between early and late pregnancy vitamin D levels and GDM, either [Early pregnancy: OR (95% CI) = 1.03 (0.95–1.06); Late pregnancy: OR (95% CI) = 1.00 (0.96–1.03)]. These observational analyses are susceptible to residual confounding and reverse causality, making it difficult to ensure a causal link of 25(OH)D level and GDM risk. Consequently, the potential preventative impact of supplementing with vitamin D during pregnancy on the development of GDM has been investigated in several randomized controlled trials (RCTs), although the findings have not been conclusive. According to a meta-analysis that included five RCT studies, supplementing with vitamin D can significantly reduce developing GDM risk [RR (95% CI) = 0.64 (0.44–0.94)] [27]. However, another RCT study from Iran showed that supplementation with 4000 IU/day of cholecalciferol starting from 8–10 gestational weeks did not reduce the risk of GDM [28]. The inconsistencies in these findings related to the type, dose, duration, and timing of vitamin D interventions, as well as differences among study participants. Additionally, the conditions of measuring and storing vitamin D can also confound the results. In conclusion, MR studies are required to provide genetic evidence for clarifying how 25(OH)D level and GDM risk are related during pregnancy.

The results of our study, however, do not point to a link between vitamin D and GDM causally. On the one hand, it is important to aware that GDM has more significant causes, such as pre-pregnancy BMI and family history of GDM, with the influence of vitamin D being relatively minor. Additionally, according to our findings, we hypothesize that vitamin D may influence the risk of GDM partly through its effect on BMI. In observational studies, the inverse association between 25(OH)D level and BMI has been well-documented [29]. In addition, a meta-analysis which included 11 RCTs revealed that supplementing with cholecalciferol reduced BMI by −0.32 kg/m^2^ (95% CI: −0.52, −0.12) [30]. The mechanisms by which vitamin D affects BMI that have been identified include regulation of parathyroid hormone and adipogenesis [31]. Overall, during pregnancy, both low vitamin D levels and high BMI contribute to increased insulin resistance and heightened inflammatory responses, ultimately promoting the development of GDM [32,33,34]. On the other hand, the overall R² of the vitamin D-related IVs is 3.357%, indicating that the variation in vitamin D is only partially explained by the IVs, and the inference of causality should be treated with caution.

The relationship between VDBP level and GDM has only been studied sporadically thus far, with inconsistent findings. Fernando et al. [35] found that lower VDBP concentration in the first trimester increased the GDM risk (OR (95% CI) = 0.98 (0.97, 0.99), N = 304), while Xia et al. [36] discovered that VDBP was not associated with the risk of GDM in either early or mid-pregnancy (all *p* > 0.05, N = 214). At the genetic level, Wang et al. [7] found an association between *GC* gene variation and GDM in Chinese pregnant women. Although our study’s findings cannot support the causal association between VDBP and GDM, we still hope that more large-scale, multi-center research will investigate the role of VDBP as an important vitamin D status indicator in pregnant women. Modulated by estrogen, VDBP level rises during pregnancy. Both vitamin D and VDBP increase during pregnancy, leading to a decrease in free vitamin D level [6]. By considering both vitamin D and VDBP to calculate free vitamin D level, the vitamin D deficiency of pregnant women can be more precisely evaluated, benefit in predicting the occurrence and development of related perinatal complications and provide guidance for pregnancy care.

This study examines the causative link between 25(OH)D, VDBP levels and GDM and is the first two-sample MR study to do so. The IVs in this study come from a meta-analysis of multiple large-sample GWAS databases, which helps to enhance the statistical power. Naturally, this study has several limitations. First, it is uncertain whether the results of this study, which was ethnicity-limited, could be applied to other ethnic communities. Second, we were unable to further explore the causative links between VDR, vitamin D deficiency or free 25(OH)D levels and GDM because pertinent SNPs were not available. VDR, whose gene and protein expression increases in placenta and decidua tissue during pregnancy, also has an important impact on vitamin D function [37]. If there are suitable public GWAS data of VDR-related SNPs, we will continue to explore the causal relationship between VDR and outcomes. Third, the vitamin D-related genetic information in this study comes from populations covering both male and female and may lack specificity for women of reproductive age. Fourth, there is a lack of consideration for other gene mutations, such as insertion, deletion, duplication, etc., but some steps in SNP screening may help reduce the impact of these mutations, such as filtering low-frequency variants. There are still few studies on the association between these gene mutations and GDM, and most of them focus only on specific individual genes and have small sample sizes [38,39,40]. We look forward to more large-sample, multi-gene explorations and discoveries on the association between these gene mutations and GDM in the future.

## 5. Conclusions

A weak negative causality of 25(OH)D and GDM risk was found in the European population, while there is little proof to support the link between VDBP and GDM. To further explore whether total or free 25(OH)D levels and GDM are causally related, GWAS data with an emphasis on women of reproductive age and other ethnic groups are required.

## Figures and Tables

**Table 1:** Introduction of datasets.

Trait	Number of Cases	Number of Controls	Sample Size	Data Source	Population	Year	ID	Fmin
Exposure								
Vitamin D	NA	NA	449,835	UKB	European	2018	ukb-d-30890_irnt	29.67
Serum 25(OH)D	NA	NA	496,946	EBI	European	2020	ebi-a-GCST90000618	30.46
25(OH)D	NA	NA	441,291	IEU	European	2020	ieu-b-4808	21.00
Vitamin D Binding Protein	NA	NA	3301	INTERVAL	European	2018	prot-a-1179	22.28
Outcome								
GDM	5687	117,892	123,579	FinnGen	European	2021	finn-b-GEST_DIABETES	/

Note: NA, not available; UKB, UK Biobank; EBI, European Bioinformatics Institute; IEU, Integrative Epidemiology Unit.

**Table 2:** 25(OH)D and GDM: two-sample Mendelian randomization studies.

Exposure	Method	SNPs	OR (95% CI)	*p*
25(OH)D	IVW	111	0.71 (0.50, 0.99)	0.046
	MR Egger	111	0.61 (0.34, 1.10)	0.103
	Maximum likelihood	111	0.70 (0.52, 0.95)	0.022
	Weighted median	111	0.72 (0.43, 1.20)	0.210
	Weighted mode	111	0.80 (0.52, 1.24)	0.314
25(OH)D (IVs independent of BMI)	IVW	90	0.76 (0.55, 1.06)	0.101
	MR Egger	90	0.66 (0.38, 1.13)	0.135
	Maximum likelihood	90	0.76 (0.55, 1.04)	0.090
	Weighted median	90	0.72 (0.43, 1.20)	0.193
	Weighted mode	90	0.77 (0.49, 1.22)	0.233

IV, instrumental variable; BMI, Body mass index.

**Table 3:** 25(OH)D and GDM: Sensitivity analyses of MR.

Exposure	Heterogeneity Test				Pleiotropy Test				
	IVW		MR–Egger		MR–Egger Intercept			MR–PRESSO Results (*p*)	
	Q	*p*	Q	*p*	Intercept	SE	*p*	Raw	Outlier-Corrected
25(OH)D	142.35	0.021	141.87	0.019	0.003	0.005	0.5465	0.0874	0.1691
25(OH)D (IVs independent of BMI)	95.01	0.312	94.54	0.298	0.003	0.005	0.5108	0.5092	NA

IV, instrumental variable; BMI, Body mass index; NA, not available.

**Table 4:** VDBP and GDM: two-sample Mendelian randomization studies.

Exposure	Method	SNPs	OR (95% CI)	*p*
VDBP	IVW	11	0.98 (0.93, 1.03)	0.408
	MR Egger	11	0.98 (0.91, 1.06)	0.680
	Maximum likelihood	11	0.98 (0.93, 1.03)	0.405
	Weighted median	11	0.98 (0.93, 1.04)	0.545
	Weighted mode	11	0.98 (0.93, 1.03)	0.473
VDBP (IVs independent of BMI)	IVW	9	0.97 (0.92, 1.02)	0.243
	MR Egger	9	0.99 (0.92, 1.06)	0.730
	Maximum likelihood	9	0.97 (0.92, 1.02)	0.248
	Weighted median	9	0.98 (0.93, 1.04)	0.527
	Weighted mode	9	0.98 (0.93, 1.03)	0.457

VDBP, vitamin D binding protein; IV, instrumental variable; BMI, Body mass index.

**Table 5:** VDBP and GDM: Sensitivity analyses of MR.

Exposure	Heterogeneity Test				Pleiotropy Test				
	IVW		MR–Egger		MR–Egger Intercept			MR–PRESSO Results (*p*)	
	Q	*p*	Q	*p*	Intercept	SE	*p*	Raw	Outlier-Corrected
VDBP	9.23	0.419	9.23	0.511	−0.003	0.015	0.8606	0.4095	NA
VDBP (IVs independent of BMI)	5.88	0.661	5.46	0.603	−0.010	0.016	0.5400	0.2103	NA

VDBP, vitamin D binding protein; IV, instrumental variable; BMI, Body mass index; NA, not available.

## Data Availability

The datasets for MR analysis of this article are available from published genome-wide association studies:
https://gwas.mrcieu.ac.uk/datasets/ukb-d-30890_irnt/ https://gwas.mrcieu.ac.uk/datasets/ebi-a-GCST90000618/ https://gwas.mrcieu.ac.uk/datasets/ieu-b-4808/ https://gwas.mrcieu.ac.uk/datasets/prot-a-1179/ https://gwas.mrcieu.ac.uk/datasets/finn-b-GEST_DIABETES/

## Supplementary Materials

The following supporting information can be downloaded at: https://www.mdpi.com/article/10.3390/nu16162603/s1, Figure S1: Leave-one-out analysis plots of MR analyses for the causal effect of 25(OH)D on GDM; Figure S2: Forest plots of MR analyses for the causal effect of 25(OH)D on GDM; Figure S3: Funnel plots of MR analyses for the causal effect of 25(OH)D on GDM; Figure S4: Leave-one-out analysis plots of MR analyses for the causal effect of VDBP on GDM; Figure S5: Forest plots of MR analyses for the causal effect of VDBP on GDM; Figure S6: Funnel plots of MR analyses for the causal effect of VDBP on GDM.
