# Supplementary material for: 25-Hydroxyvitamin D, Vitamin D Binding Protein and Gestational Diabetes Mellitus: A Two-Sample Mendelian Randomization Study
Source: Nutrients. 2024 Aug 7;16(16):2603. doi: 10.3390/nu16162603 (PMC11356852; doi:10.3390/nu16162603)
Supplement: Supplementary file 1 [file nutrients-16-02603-s001.zip › Supplementary figure legends.pdf]

Supplementary figure legends

Figure S1. Leave-one-out analysis plots of MR analyses for the causal effect of 25(OH)D on GDM

(A) Analysis of 25(OH)D and GDM.

(B) Analysis of 25(OH)D and GDM with IVs independent of BMI.

MR, mendelian randomization; GDM, gestational diabetes mellitus; IV, instrumental variable; BMI_Independent, selected IVs independent of BMI.

Figure S2. Forest plots of MR analyses for the causal effect of 25(OH)D on GDM

(A) Analysis of 25(OH)D and GDM.

(B) Analysis of 25(OH)D and GDM with IVs independent of BMI.

MR, mendelian randomization; GDM, gestational diabetes mellitus; IV, instrumental variable; BMI_Independent, selected IVs independent of BMI.

Figure S3. Funnel plots of MR analyses for the causal effect of 25(OH)D on GDM

(A) Analysis of 25(OH)D and GDM.

(B) Analysis of 25(OH)D and GDM with IVs independent of BMI.

MR, mendelian randomization; GDM, gestational diabetes mellitus; IV, instrumental variable; BMI_Independent, selected IVs independent of BMI.

Figure S4. Leave-one-out analysis plots of MR analyses for the causal effect of VDBP on GDM

(A) Analysis of VDBP and GDM.

(B) Analysis of VDBP and GDM with IVs independent of BMI.

MR, mendelian randomization; VDBP, vitamin D binding protein; GDM, gestational diabetes mellitus; IV, instrumental variable; BMI_Independent, selected IVs independent of BMI.

Figure S5. Forest plots of MR analyses for the causal effect of VDBP on GDM

(A) Analysis of VDBP and GDM.

(B) Analysis of VDBP and GDM with IVs independent of BMI.

MR, mendelian randomization; VDBP, vitamin D binding protein; GDM, gestational diabetes mellitus; IV, instrumental variable; BMI_Independent, selected IVs independent of BMI.

Figure S6. Funnel plots of MR analyses for the causal effect of VDBP on GDM

(A) Analysis of VDBP and GDM.

(B) Analysis of VDBP and GDM with IVs independent of BMI.

MR, mendelian randomization; VDBP, vitamin D binding protein; GDM, gestational diabetes mellitus; IV, instrumental variable; BMI_Independent, selected IVs independent of BMI.

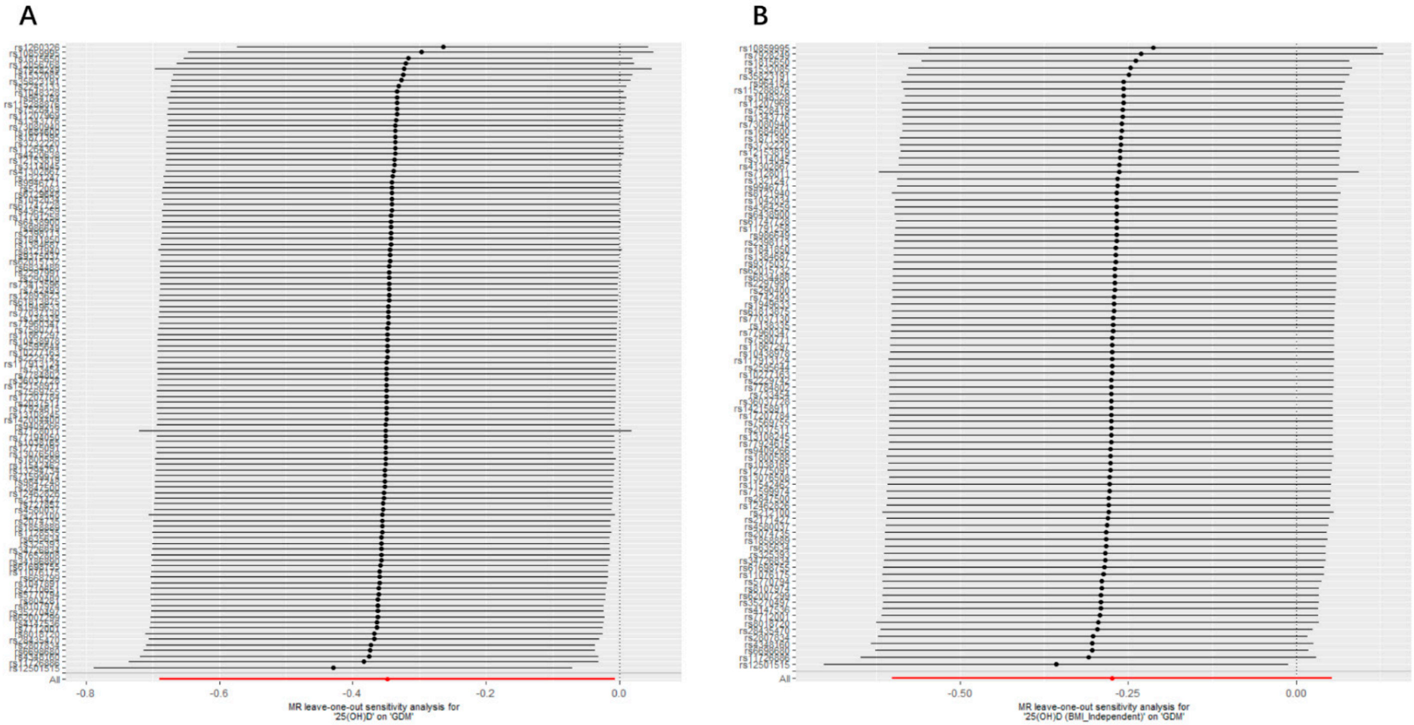

Figure S1. Leave-one-out analysis plots of MR analyses for the causal effect of 25(OH)D on GDM

(A) Analysis of 25(OH)D and GDM. (B) Analysis of 25(OH)D and GDM with IVs independent of BMI. MR, mendelian randomization; GDM, gestational diabetes mellitus; IV, instrumental variable; BMI_Independent, selected IVs independent of BMI.

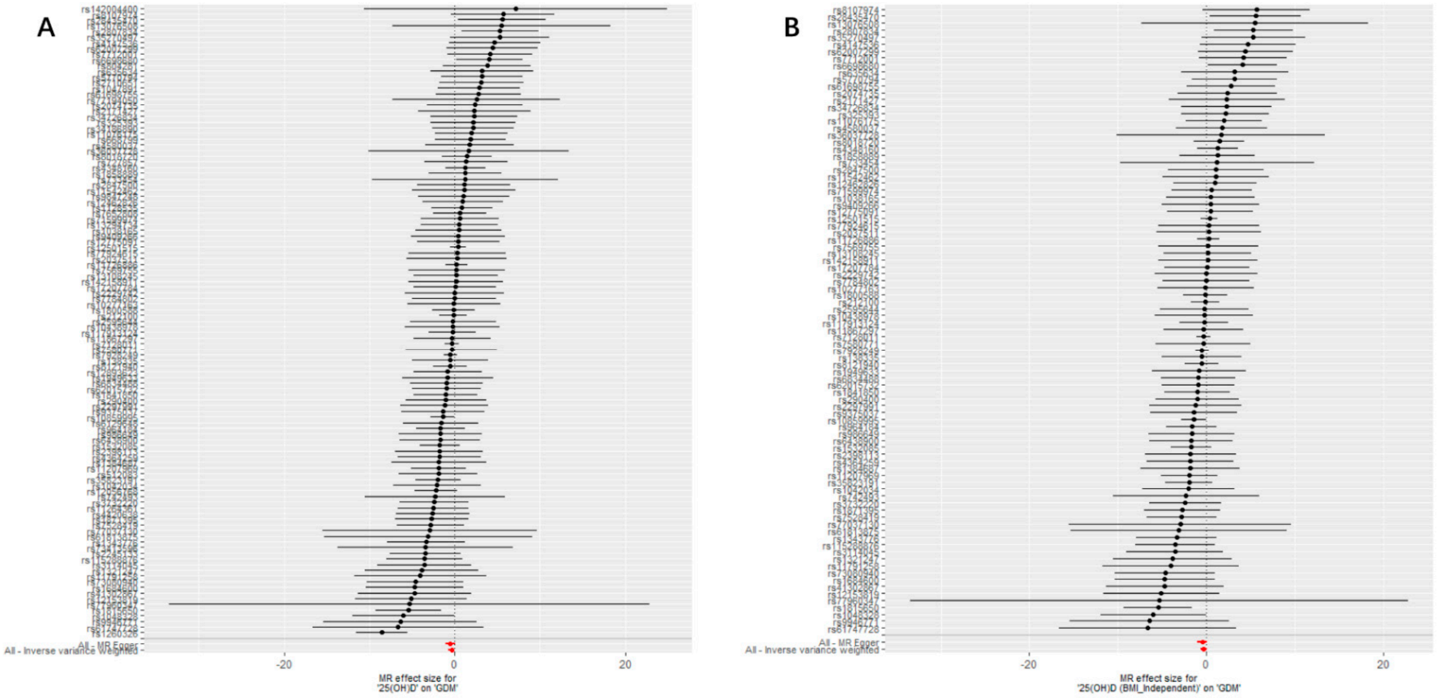

Figure S2. Forest plots of MR analyses for the causal effect of 25(OH)D on GDM

(A) Analysis of 25(OH)D and GDM. (B) Analysis of 25(OH)D and GDM with IVs independent of BMI. MR, mendelian randomization; GDM, gestational diabetes mellitus; IV, instrumental variable; BMI_Independent, selected IVs independent of BMI.

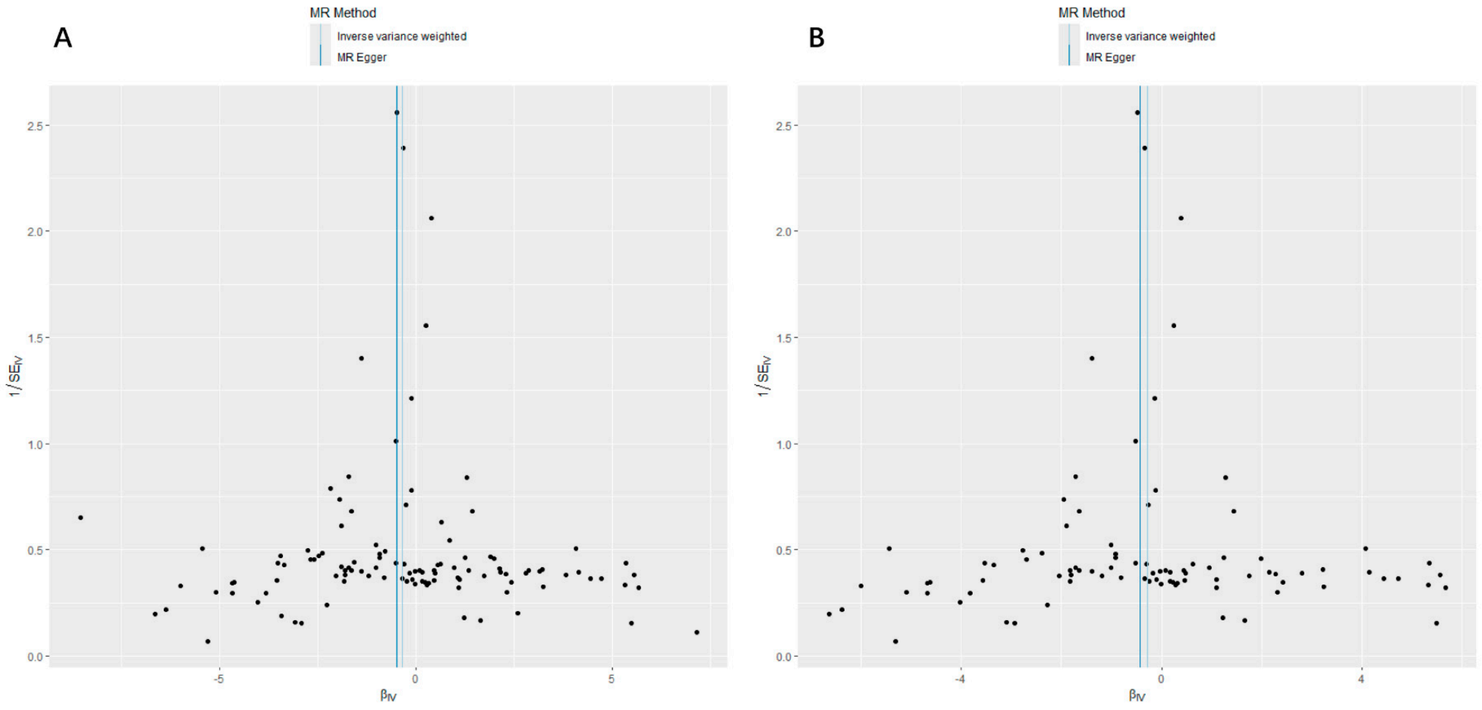

Figure S3. Funnel plots of MR analyses for the causal effect of 25(OH)D on GDM

(A) Analysis of 25(OH)D and GDM. (B) Analysis of 25(OH)D and GDM with IVs independent of BMI. MR, mendelian randomization; GDM, gestational diabetes mellitus; IV, instrumental variable; BMI_Independent, selected IVs independent of BMI.

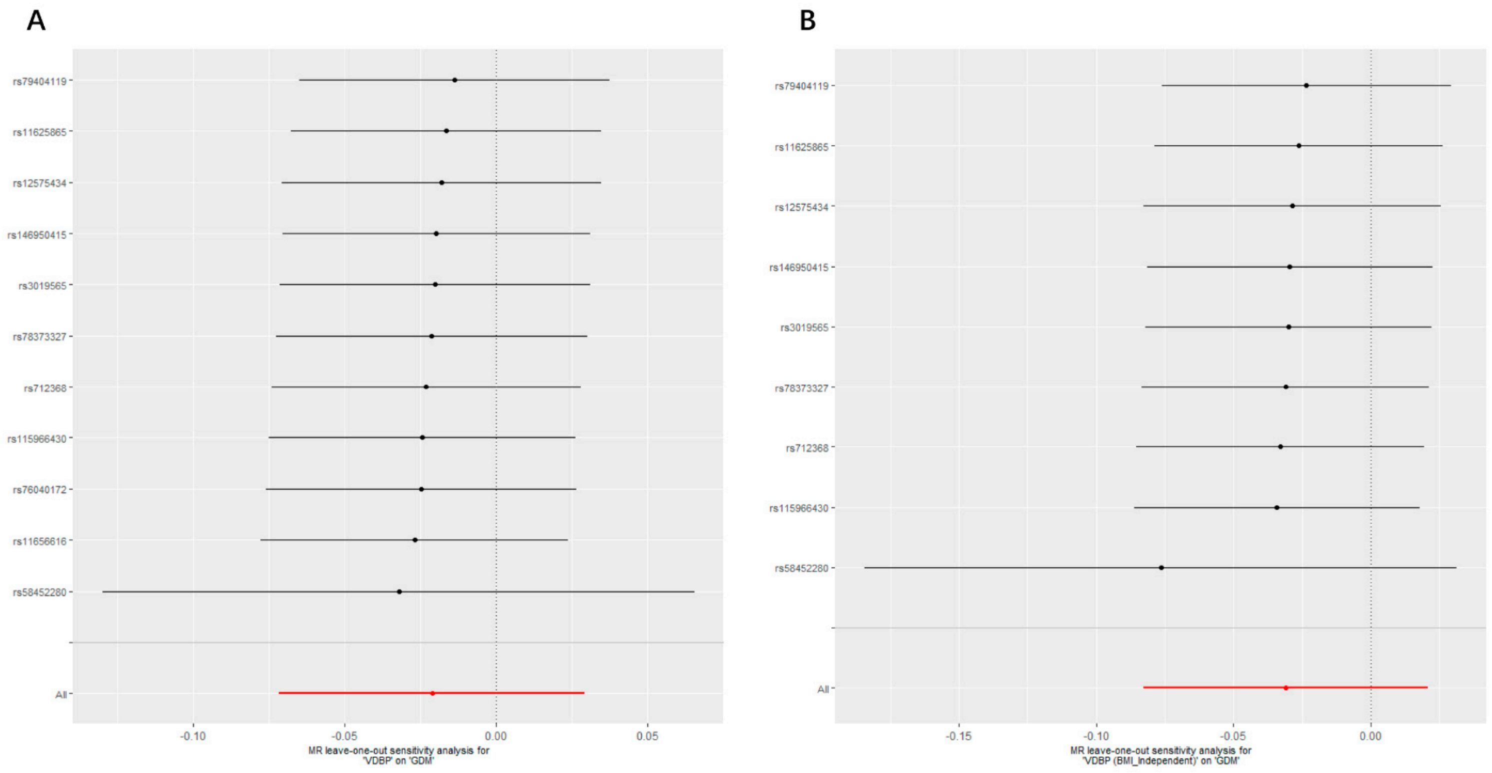

Figure S4. Leave-one-out analysis plots of MR analyses for the causal effect of VDBP on GDM

(A) Analysis of VDBP and GDM. (B) Analysis of VDBP and GDM with IVs independent of BMI. MR, mendelian randomization; VDBP, vitamin D binding protein; GDM, gestational diabetes mellitus; IV, instrumental variable; BMI_Independent, selected IVs independent of BMI.

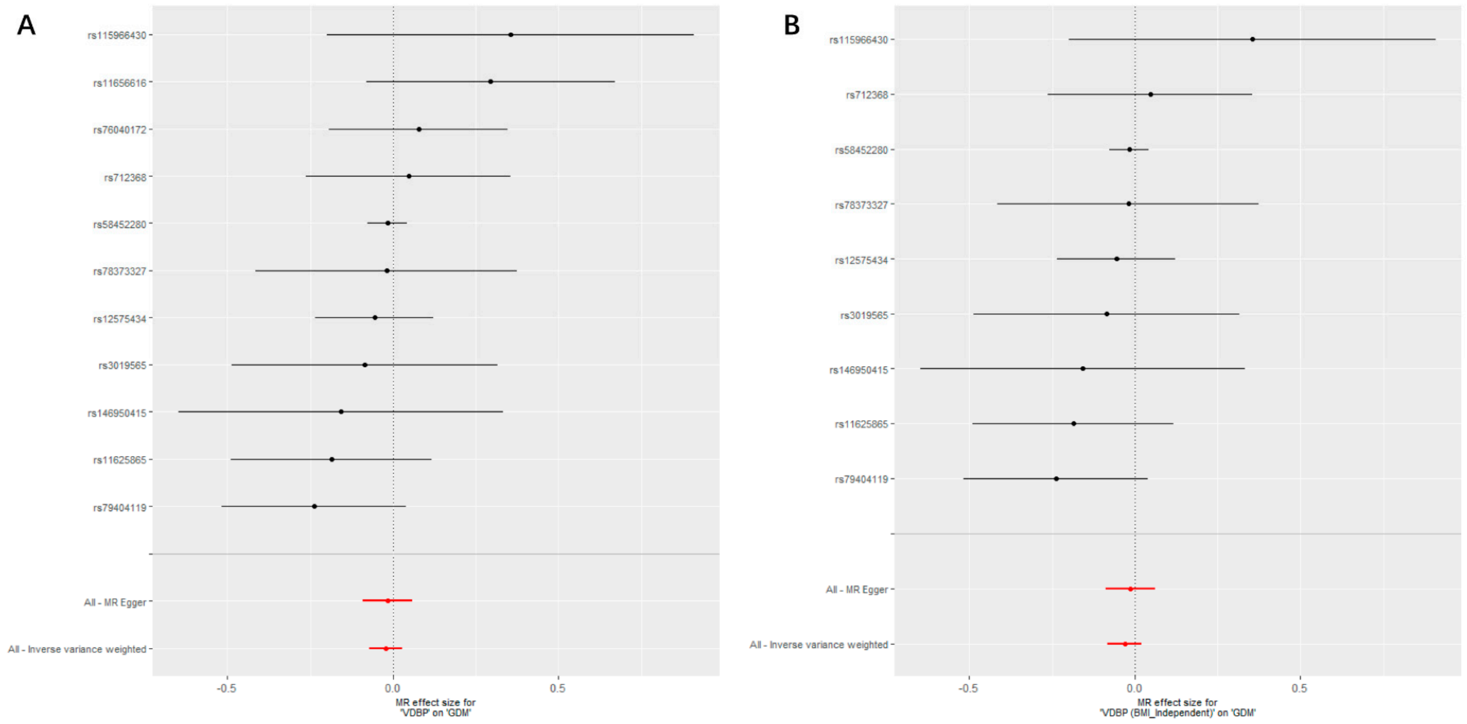

Figure S5. Forest plots of MR analyses for the causal effect of VDBP on GDM

(A) Analysis of VDBP and GDM. (B) Analysis of VDBP and GDM with IVs independent of BMI. MR, mendelian randomization; VDBP, vitamin D binding protein; GDM, gestational diabetes mellitus; IV, instrumental variable; BMI_Independent, selected IVs independent of BMI.

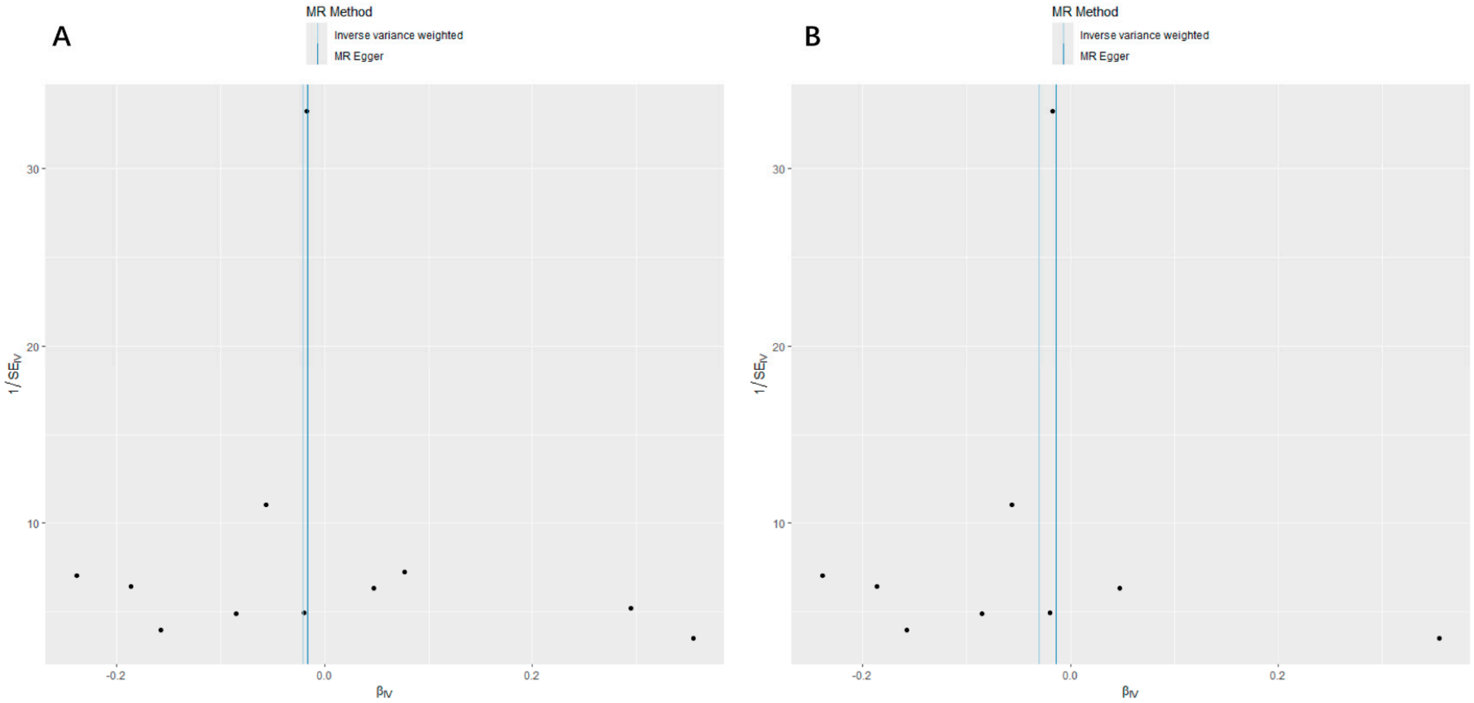

Figure S6. Funnel plots of MR analyses for the causal effect of VDBP on GDM

(A) Analysis of VDBP and GDM. (B) Analysis of VDBP and GDM with IVs independent of BMI. MR, mendelian randomization; VDBP, vitamin D binding protein; GDM, gestational diabetes mellitus; IV, instrumental variable; BMI_Independent, selected IVs independent of BMI.
