# Supplementary figures and images for: 25-Hydroxyvitamin D, Vitamin D Binding Protein and Gestational Diabetes Mellitus: A Two-Sample Mendelian Randomization Study
Source: Nutrients. 2024 Aug 7;16(16):2603. doi: 10.3390/nu16162603 (PMC11356852; doi:10.3390/nu16162603)

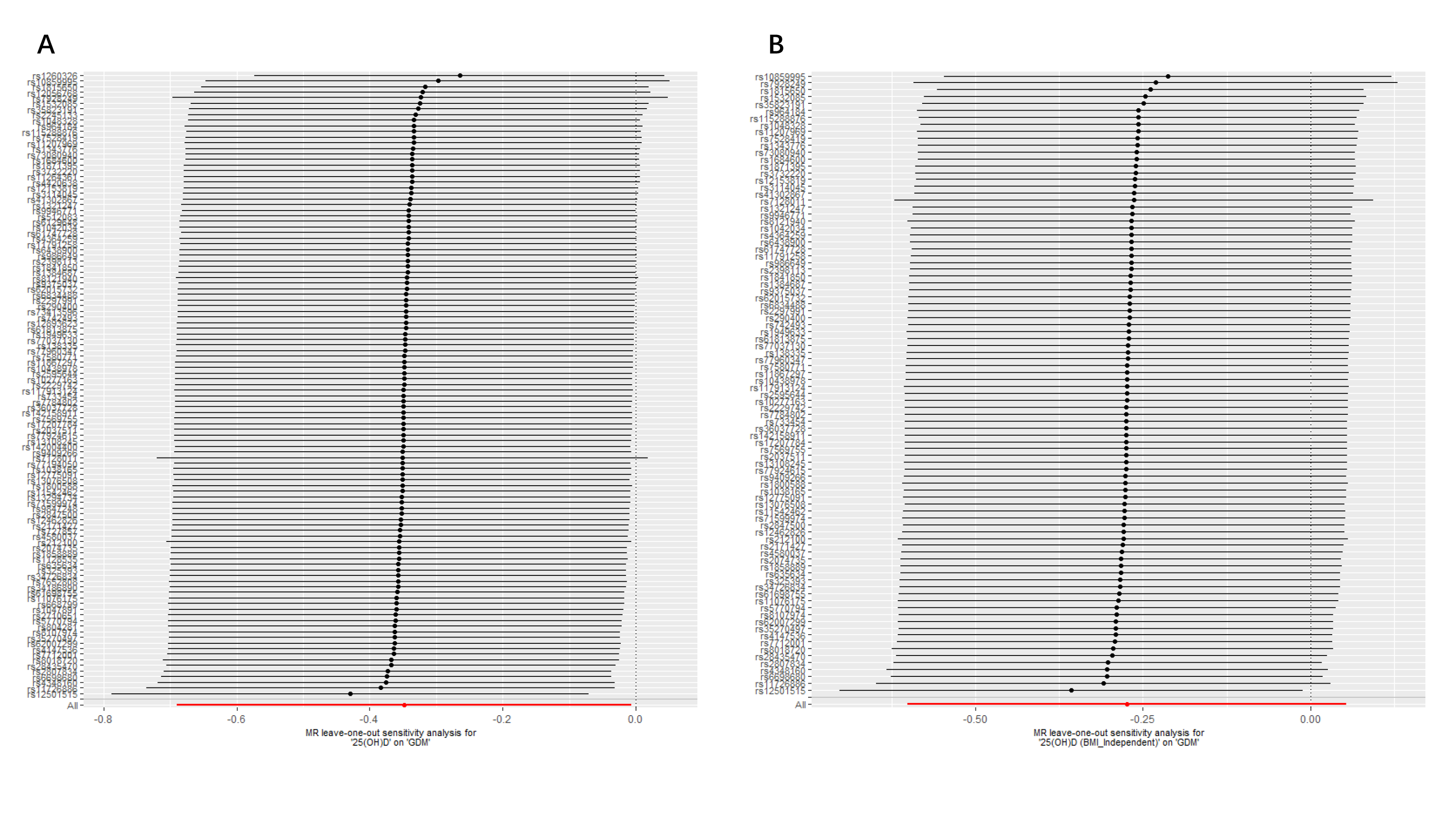

Supplement: Supplementary file 1 [file nutrients-16-02603-s001.zip › Figure S1.tif]

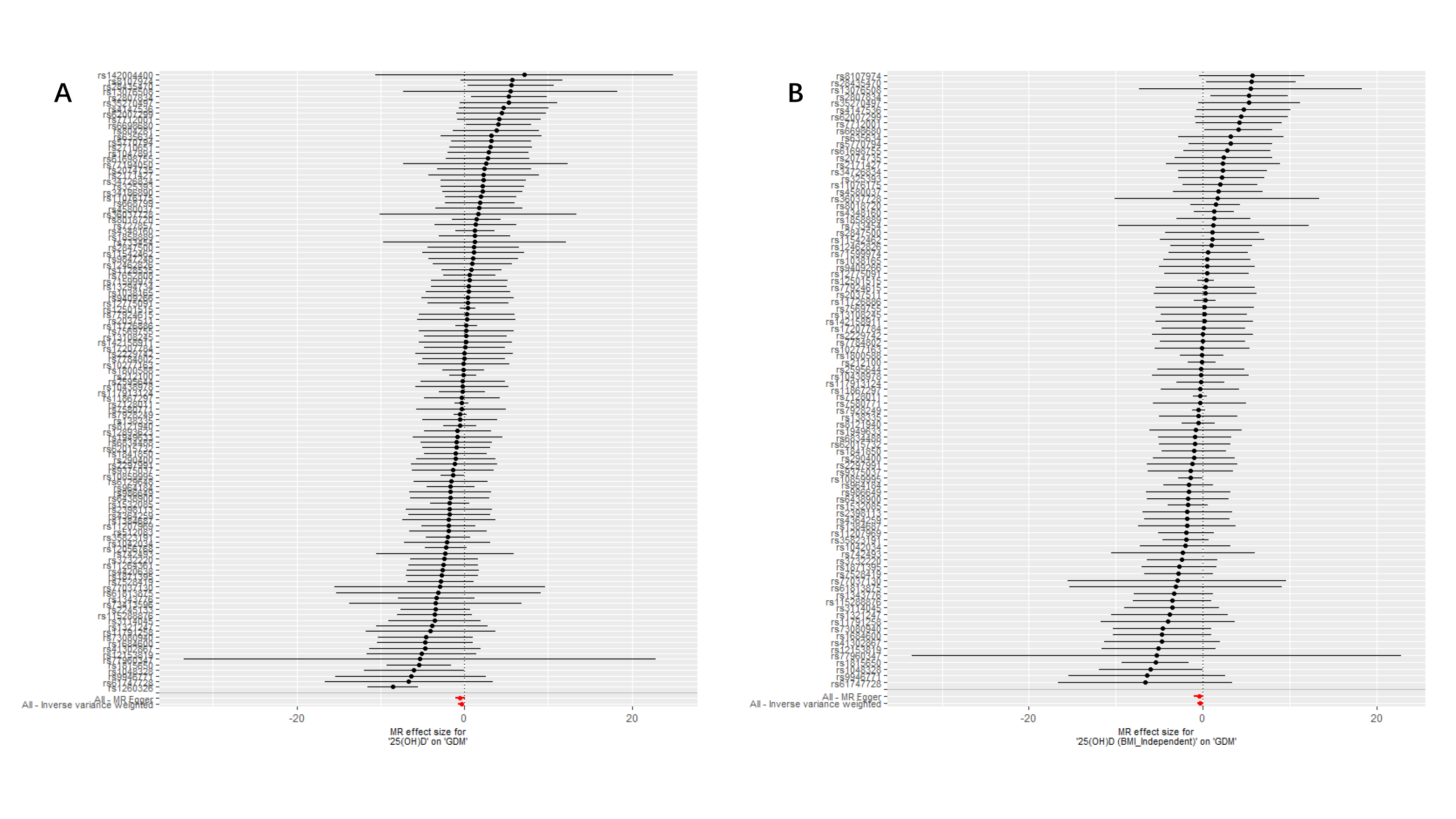

Supplement: Supplementary file 1 [file nutrients-16-02603-s001.zip › Figure S2.tif]

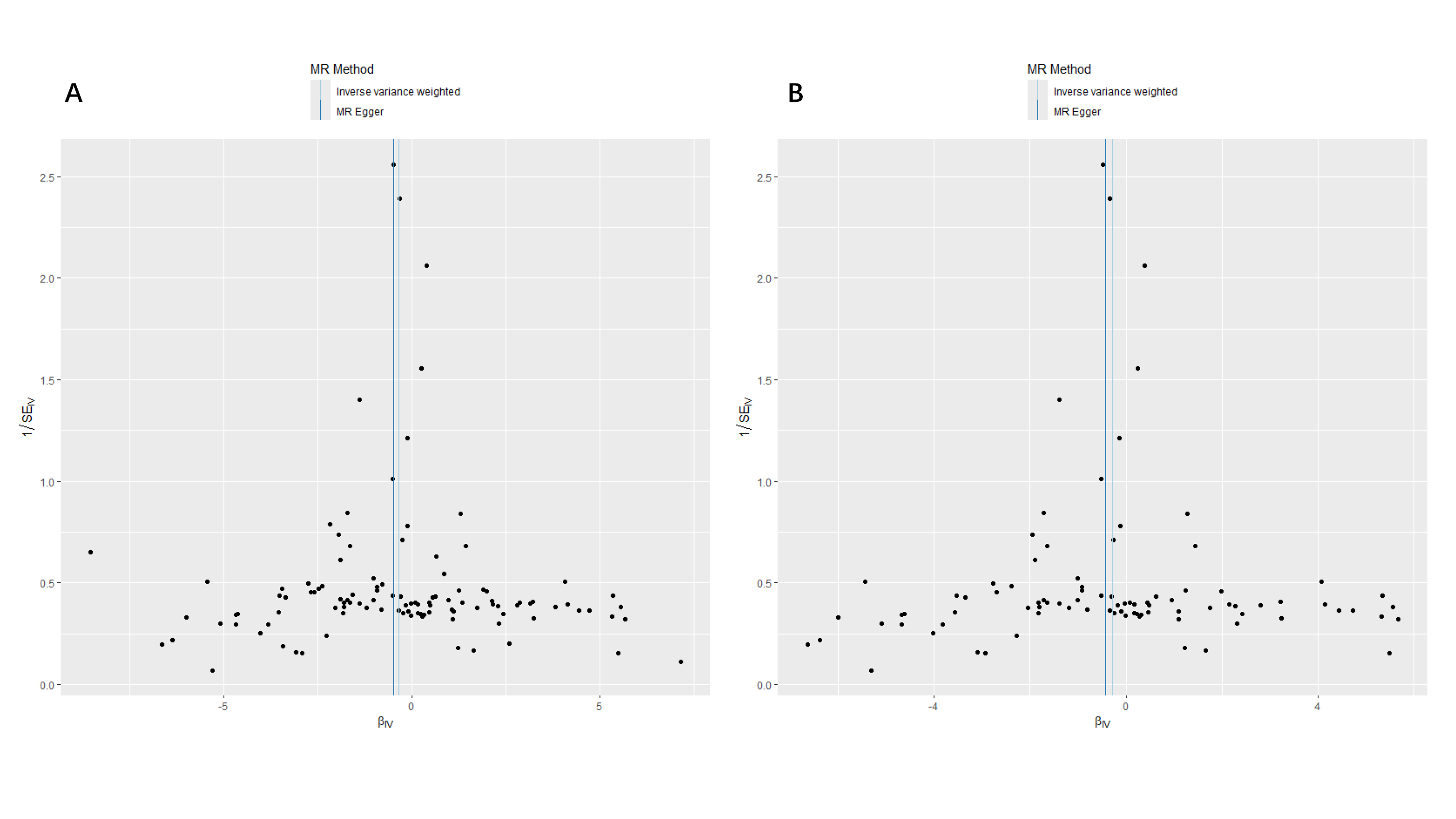

Supplement: Supplementary file 1 [file nutrients-16-02603-s001.zip › Figure S3.tif]

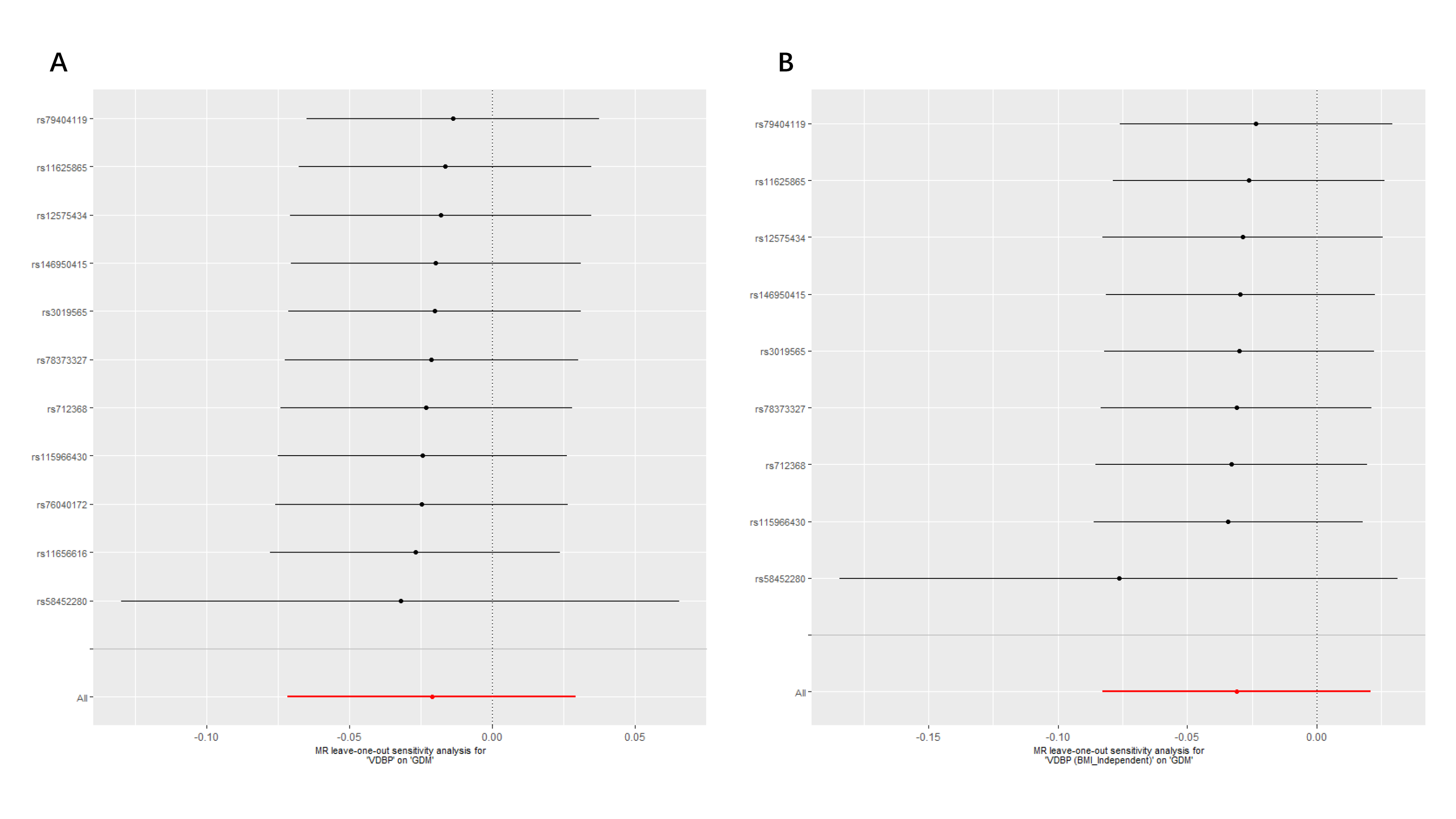

Supplement: Supplementary file 1 [file nutrients-16-02603-s001.zip › Figure S4.tif]

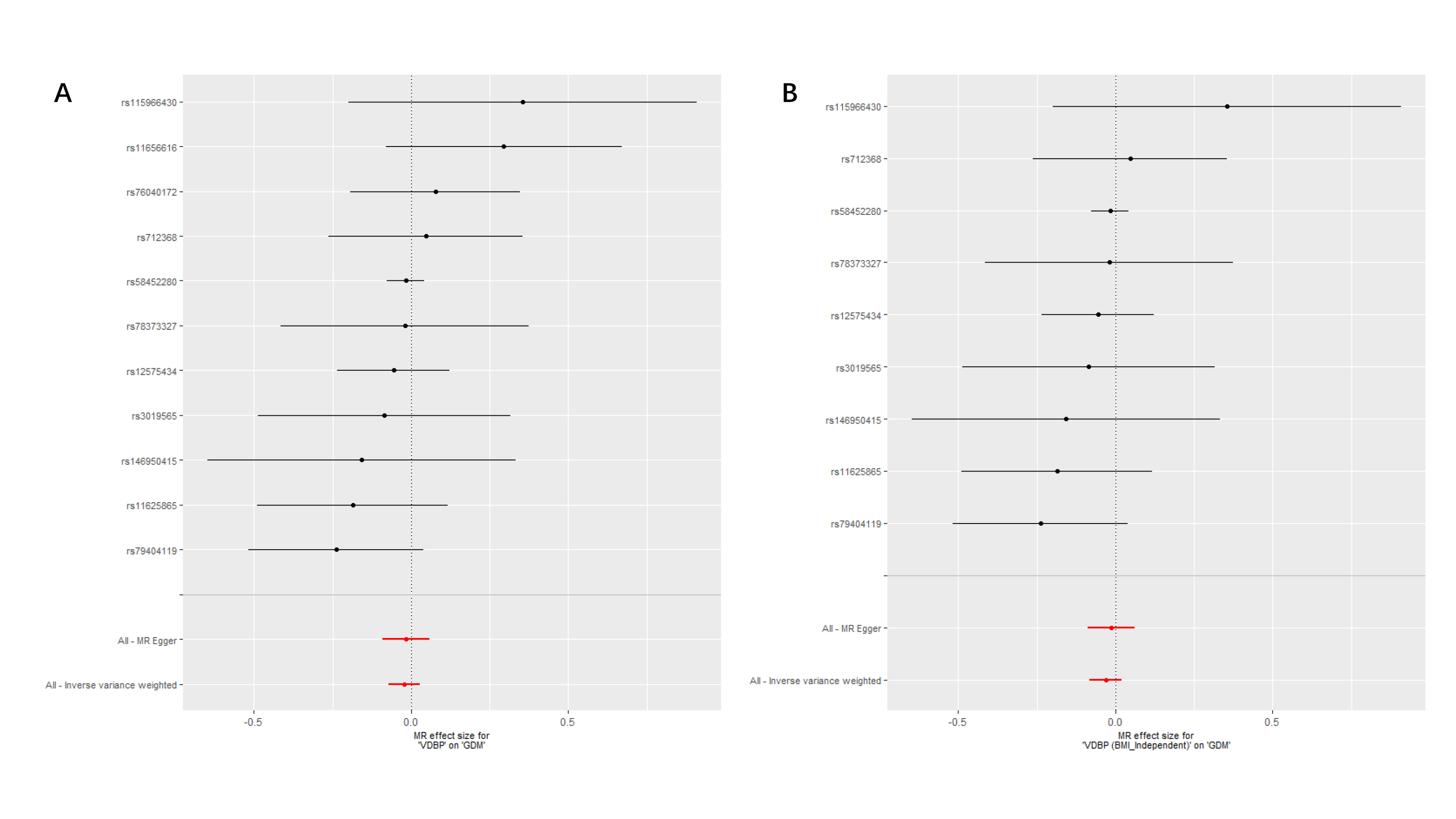

Supplement: Supplementary file 1 [file nutrients-16-02603-s001.zip › Figure S5.tif]

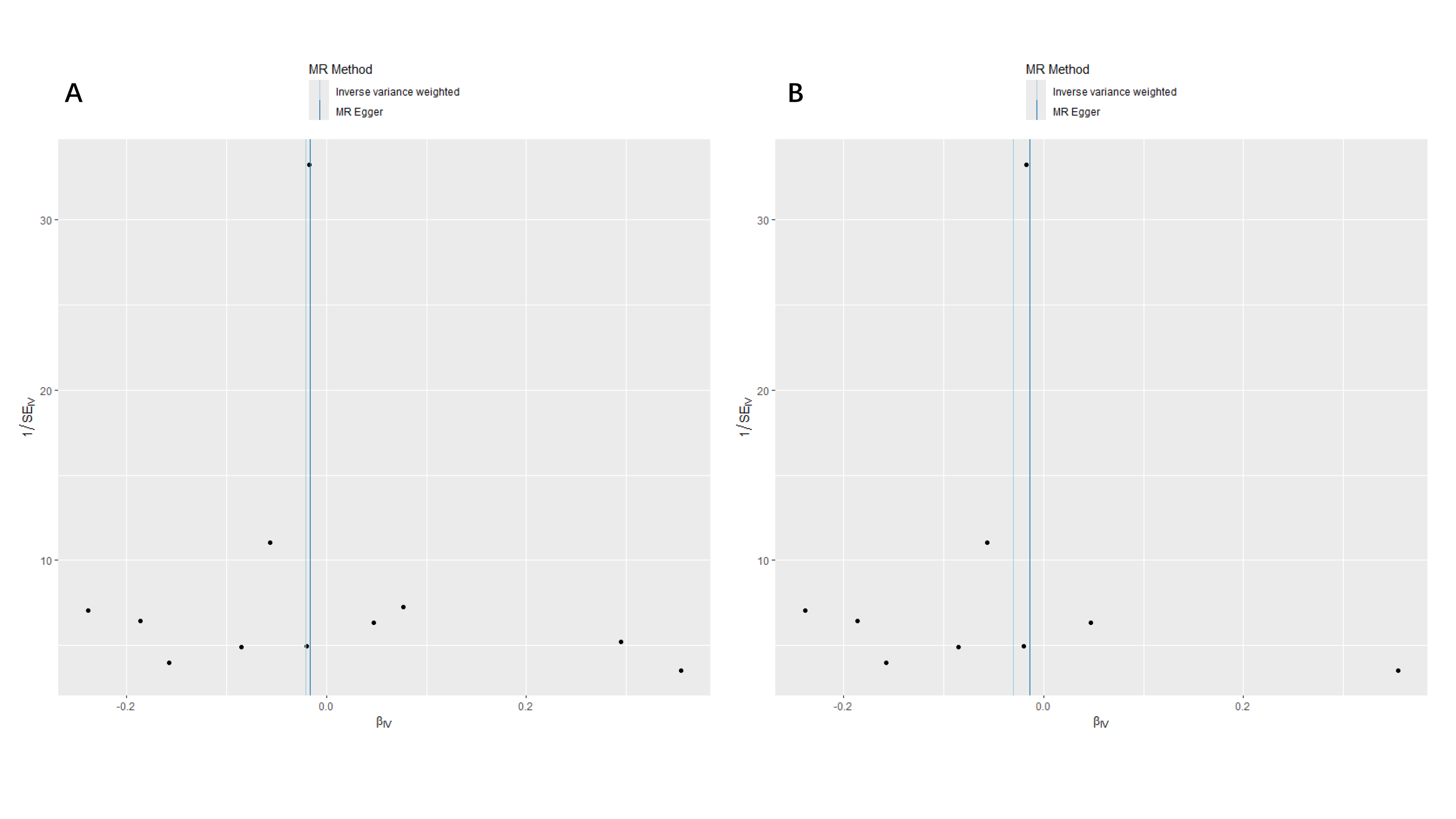

Supplement: Supplementary file 1 [file nutrients-16-02603-s001.zip › Figure S6.tif]
